# Total Muscle Area and Visceral Adipose Tissue Measurements for Frailty Assessment in TAVR Patients

**DOI:** 10.3390/jcm13051322

**Published:** 2024-02-26

**Authors:** Caglayan Demirel, Christoph Fritz Rothenbühler, Markus Huber, Michelle Schweizer, Inga Todorski, David Alexander Gloor, Stephan Windecker, Jonas Lanz, Stefan Stortecky, Thomas Pilgrim, Gabor Erdoes

**Affiliations:** 1 Department of Cardiology, Inselspital, University of Bern, 3012 Bern, Switzerland; caglayan.demirel@meduniwien.ac.at (C.D.); stephan.windecker@insel.ch (S.W.); jonas.lanz@insel.ch (J.L.); stefan.stortecky@insel.ch (S.S.); thomas.pilgrim@insel.ch (T.P.); 2 Department of Anaesthesiology and Pain Medicine, Inselspital, University of Bern, 3012 Bern, Switzerland; christophfritz.rothenbuehler@insel.ch (C.F.R.); markus.huber@insel.ch (M.H.); 3 Department of Radiology, Inselspital, University of Bern, 3012 Bern, Switzerland; michelle.schweizer@insel.ch (M.S.); inga.todorski@insel.ch (I.T.); davidalexander.gloor@insel.ch (D.A.G.)

**Keywords:** aortic valve stenosis, body composition parameters, CT scan, sarcopenia, frailty, mortality

## Abstract

**Background:** Transcatheter aortic valve replacement (TAVR) is a treatment option for severe aortic valve stenosis. Pre-TAVR assessments, extending beyond anatomy, include evaluating frailty. Potential frailty parameters in pre-TAVR computed tomography (CT) scans are not fully explored but could contribute to a comprehensive frailty assessment. The primary objective was to investigate the impact of total muscle area (TMA) and visceral adipose tissue (VAT) as frailty parameters on 5-year all-cause mortality in patients undergoing TAVR. **Methods:** Between 01/2017 and 12/2018, consecutive TAVR patients undergoing CT scans enabling TMA and VAT measurements were included. **Results:** A total of 500 patients qualified for combined TMA and VAT analysis. Age was not associated with a higher risk of 5-year mortality (HR 1.02, 95% CI: 0.998–1.049; *p* = 0.069). Body surface area normalized TMA (nTMA) was significantly associated with 5-year, all-cause mortality (HR 0.927, 95% CI: 0.927–0.997; *p* = 0.033), while VAT had no effect (HR 1.002, 95% CI: 0.99–1.015; *p* = 0.7). The effect of nTMA on 5-year, all-cause mortality was gender dependent: the protective effect of higher nTMA was found in male patients (p_interaction_: sex × nTMA = 0.007). **Conclusions:** Normalized total muscle area derived from a routine CT scan before transcatheter aortic valve replacement complements frailty assessment in patients undergoing TAVR.

## 1. Introduction

Due to significant advancements in transcatheter aortic valve replacement (TAVR) safety, efficacy, and noninferiority compared to surgical aortic valve replacement (SAVR), the number of TAVR procedures is rising [1,2,3,4,5,6].

A detailed scrutiny of individual patient data, conducted via a meta-analysis derived from randomized clinical trials, offers a nuanced comparison between TAVR and SAVR. The comprehensive evaluation conducted at the one-year milestone discerns a distinct advantage for TAVR, manifesting as a lower risk of death, disabling stroke, and the composite endpoint of death or disabling stroke, positioning TAVR as a compelling alternative to SAVR [5].

Delving deeper into the intricate landscape of clinical outcomes, a longitudinal study focused on patients with severe aortic stenosis and intermediate surgical risk postulates no significant disparity in the incidence of death or disabling stroke at the five-year juncture between TAVR and SAVR [6].

This corroborates the findings from a randomized trial involving intermediate-risk patients, emphasizing the parallel nature of TAVR and SAVR in terms of the primary endpoint of death or disabling stroke [4].

Expanding the investigational lens to encompass low-risk patient cohorts, a seminal antecedent study substantiates the non-inferiority of TAVR featuring a self-expanding supra-annular bioprosthesis relative to surgery. Notably, this comparison centers on the composite endpoint of death or disabling stroke at the 24-month juncture, further establishing TAVR’s efficacy in this specific patient demographic [2].

Regarding balloon-expandable valves within low-risk patients, TAVR exhibits a significantly lower rate of the composite endpoint, encompassing death, stroke, or rehospitalization, at the one-year mark, underscoring its superiority over surgical alternatives [1].

Collectively, these multifaceted findings contribute substantially to the evolving narrative surrounding TAVR’s efficacy. They underscore its favorable outcomes across a spectrum of risk profiles, solidifying its standing as a credible and viable alternative to conventional surgical approaches in nuanced clinical scenarios. This nuanced understanding aids in the refinement of patient-centered treatment strategies and reinforces the role of TAVR as a pivotal intervention in contemporary cardiovascular care.

In a contemporary investigation, the projected incidence rates of transcatheter aortic valve replacement and surgical aortic valve replacement demonstrated notable variability, spanning from 12 to 22 and 20 to 35 cases per 100,000 individuals in Switzerland, respectively. These variations were observed within diverse hospital service areas across Switzerland. This study sheds light on the geographic heterogeneity in the utilization of these interventions, highlighting the influence of regional factors on the prevalence of TAVR and SAVR procedures within the Swiss healthcare landscape. Such nuanced insights contribute to a more comprehensive understanding of the healthcare dynamics and resource allocation patterns related to aortic valve replacement strategies in the country [7].

The presented data accentuate the paramount importance of conducting an exhaustive pre- TAVR workup. Beyond the routine evaluation of anatomical parameters, such as femoral access size, valve type and size considerations, and an assessment of coronary status, this study underscores the pivotal role of incorporating broader health metrics into the preparatory phase.

An optimal pre-TAVR workup recognizes the multifaceted nature of patient health, extending the evaluation beyond the confines of structural considerations to encompass a comprehensive examination of a general medical condition and its frailty parameters. 

The inclusion of parameters related to general medical condition and frailty in the pre-TAVR evaluation process is essential for several reasons. Firstly, it allows for a more holistic understanding of the patient’s overall health, providing insight into potential challenges and optimizing the decision-making process. Secondly, this approach aligns with the evolving paradigm of personalized medicine, emphasizing the customization of medical interventions based on individual patient characteristics.

A comprehensive pre-TAVR workup that encompasses a detailed assessment of a general medical condition and frailty not only enhances the procedural planning but also contributes to the establishment of a baseline for post-procedural care and rehabilitation. This patient-centered approach is crucial to optimizing outcomes, reducing complications, and improving the overall quality of care in the realm of transcatheter interventions for aortic valve replacement.

The process of measuring anatomical structures has been standardized and enhanced with precision through the utilization of TAVR computed tomography (CT) scans. However, the assessment of frailty, a critical determinant in patient outcomes, poses a more challenging task due to its subjective and multifaceted nature, lacking a universally accepted standard for evaluation. While tools like the Society of Thoracic Surgeons (STS) and European System for Cardiac Operative Risk Evaluation (EuroSCORE)-2 scores have become integral components of heart team evaluations for outcome prediction in TAVR, there exists a compelling need for a more nuanced and comprehensive evaluation of frailty.

Frailty, characterized by reduced physiological reserves and an increased vulnerability to stressors, is a complex concept that encompasses various dimensions, including physical, cognitive, and psychosocial aspects. Despite its recognized significance in predicting surgical outcomes, achieving consensus on a standardized frailty assessment tool remains elusive within the medical community.

The current reliance on scores such as STS and EuroSCORE-2, while valuable, may not fully capture the intricacies of frailty, which extends beyond the purely physiological [8]. A holistic evaluation that encompasses not only the anatomical considerations provided by TAVR CT scans but also a detailed assessment of frailty is paramount. This is particularly relevant as frailty has been identified as an independent predictor of adverse outcomes in TAVR procedures.

Previous studies have analyzed factors such as age, cognitive impairment, physical weakness, and also laboratory values such as anemia and hypoalbuminemia [9]. There is a great heterogeneity in clinical vulnerability, including nutritional status, physical and cognitive impairment, and psychosocial risk factors [10].

In the pursuit of a more comprehensive understanding of patient suitability for TAVR, efforts should be directed towards the incorporation of validated frailty assessment tools into routine clinical practice. By integrating these measures, the heart team can achieve a more nuanced risk stratification, better anticipate potential challenges, and tailor interventions to the unique needs of each patient. As the field of transcatheter interventions continues to evolve, the refinement of frailty assessment methodologies stands out as an imperative stride towards enhancing the precision and individualization of cardiovascular care.

In the quest for objective frailty parameters, previous studies have delved into the analysis of body composition parameters, focusing specifically on two pivotal tissues: muscle mass and fat mass. These parameters extend beyond mere cardiac risk assessment and encompass a broader spectrum by being evaluated for noncardiac risk assessment as well.

The examination of muscle mass and fat mass as objective markers of frailty introduces a comprehensive dimension to risk assessment protocols. Traditionally, frailty assessments have often centered around physiological and functional domains, but the inclusion of body composition parameters adds a more tangible and quantifiable aspect to the evaluation process. This approach recognizes the systemic nature of frailty, acknowledging that alterations in body composition can have profound implications for overall health and resilience.

The incorporation of muscle mass as a parameter is particularly pertinent, as it reflects not only on the individual’s physical strength but also on their capacity to withstand stressors and recover from interventions. Conversely, the assessment of fat mass acknowledges the role of adipose tissue in metabolic and inflammatory processes, providing insights into the overall metabolic health of the patient.

By extending the analysis of body composition parameters to a noncardiac risk assessment, these studies broaden the scope of frailty evaluation. Recognizing that frailty is a holistic concept, affected by both cardiac and noncardiac factors, allows for a more encompassing risk stratification strategy. This holistic approach aligns with the evolving understanding of frailty as a multifaceted entity and emphasizes the need for a comprehensive evaluation that transcends traditional boundaries [11,12,13,14,15,16,17].

As the field advances, the integration of body composition parameters into frailty assessments not only enriches risk prediction models but also opens avenues for tailored interventions. The nuanced insights gained from evaluating muscle and fat mass contribute to a more refined understanding of the patient’s overall health, facilitating personalized care strategies that extend beyond the confines of cardiac considerations.

With respect to muscle mass, studies have shown that sarcopenia is associated with frailty in the elderly population [18]. For example, quadriceps depth measured by ultrasound was a predictor of adverse postoperative outcomes, including discharge to a skilled nursing facility and delirium [19]. Measurement of sarcopenia using the psoas muscle area (PMA) has also been evaluated [20,21]. Studies have been performed with regard to subcutaneous and visceral adipose tissue fat mass, analyzing the effects on noncardiac and cardiac perioperative outcomes and survival [22,23].

In summary, data are available for both muscle mass and visceral adipose tissue (VAT) to support them as predictors of poor outcome after surgery.

In the realm of assessing muscle mass in the context of patients undergoing TAVR, the predominant focus has been on measuring the PMA. However, a notable gap exists in the available data concerning the effects of TMA, a comprehensive measure that considers the entire muscle mass within a given segment. This limitation extends to an unclear understanding of how the interplay between TMA and VAT influences outcomes post-TAVR.

Motivated by this knowledge gap, our research endeavors aim to delve into the intricacies of muscle mass evaluation in the TAVR setting. Our primary objective is to identify an objective marker of frailty within this patient population. We hypothesize that a diminished TMA will be associated with poorer survival outcomes post-TAVR, signifying the potential significance of overall muscle mass in predicting patient prognosis.

Furthermore, we posit a secondary hypothesis, postulating that the combined assessment of low TMA and low VAT may yield a more robust predictive marker for adverse outcomes in TAVR patients. This dual evaluation seeks to explore potential synergies or interactions between total muscle area and visceral adipose tissue, offering a more comprehensive understanding of the factors influencing patient outcomes beyond individual muscle mass components.

Through this nuanced exploration of muscle mass and its interplay with visceral adipose tissue, our study aspires to contribute valuable insights into refining risk stratification models and enhancing prognostic markers for patients undergoing TAVR. By elucidating the intricate relationships between these parameters, we aim to pave the way for more targeted and personalized interventions in the pursuit of improved outcomes for TAVR patients.

## 2. Methods

### 2.1. Study Design and Population

All patients undergoing TAVR at our university-based tertiary referral center are consecutively recorded in a prospective institutional database as part of the Swiss TAVI registry, which is mandated by the national health authorities (registered at clinicaltrials.gov with NCT01368250) [24]. The present analysis is retrospective and encompasses all individuals diagnosed with severe aortic valve stenosis who underwent transfemoral (TF)-TAVR using contemporary balloon-expandable devices (SAPIEN 3, SAPIEN 3 Ultra [Edwards Lifesciences, Irvine, CA, USA]) or self-expanding devices (Evolut R/PRO [Medtronic, Minneapolis, MN, USA]) from January 2017 to December 2018.

Our study encompassed all patients (*n* = 604) who underwent TAVR between January 2017 and December 2018. However, due to constraints in image cropping, combined measurements of TMA and VAT were only possible in 500 patients, leading to their exclusion, representing 82.8% of the overall patient cohort during the study period.

The registry was approved by the local ethics committee. All patients provided written informed consent for participation.

### 2.2. Transcatheter Aortic Valve Replacement

The TAVR procedures via the transfemoral route were executed in adherence to institutional protocols, conducted either under local or general anesthesia within a hybrid catheterization laboratory setting. Consistent with prevailing guidelines, oral anti-coagulation was deliberately ceased in the lead up to the TAVR procedure. Throughout the TAVR intervention, patients were administered unfractionated heparin with a predefined target activated clotting time of 250 s. Concurrently, any ongoing antiplatelet therapy was maintained in accordance with the patient’s pre-existing regimen. This standardized approach aligns with the established best practices in the field, reflecting a commitment to procedural precision and patient safety during the transcatheter aortic valve implantation process.

### 2.3. CT Assessment

**Figure 1:**
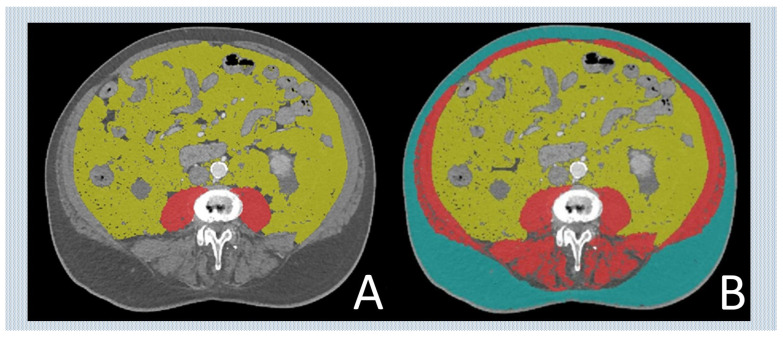
Measurement of each body composition parameter, total muscle area (TMA) and visceral adipose tissue (VAT). Legend (**A**): PMA (psoas muscle area) marked in red, SAT (subcutaneous adipose tissue) indicated in turquoise; Legend (**B**): TMA (total muscle area) marked in red, VAT (visceral adipose tissue) highlighted in yellow, and SAT (subcutaneous adipose tissue) indicated in turquoise.

All patients who had a CT scan before TAVR were included. A volumetric assessment of each CT scan was performed. Here, a single axial image was acquired at the level of the middle third lumbar vertebra (L3) and analyzed with dedicated software (Sliceomatic, TomoVision v5.0, 3280 chemin Milletta, Magog, QC, Canada) by measuring VAT area (cm^2^) and TMA. (Figure 1) TMA was normalized by division with body surface area. VAT was measured in cm^2^.

### 2.4. Data Collection

Baseline clinical, procedural, and follow-up data were prospectively recorded in a web-based database held at the Clinical Trials Unit of the University of Bern. Clinical follow up was obtained from standardized telephone interviews, medical reports, and hospital discharge summaries. All adverse events were independently adjudicated by a clinical event committee according to the Valve Academic Research Consortium (VARC) criteria applicable at the time of the procedure at the University of Bern [25].

### 2.5. Endpoints

The primary objective endpoint of this analysis was to assess the impact of normalized TMA (nTMA) and VAT on 5-year, all-cause mortality.

### 2.6. Statistical Analysis

Categorical variables are presented as frequencies and percentages. Continuous variables are presented as mean and standard deviation if they are normally distributed variables and are presented with median and interquartile range (IQR) otherwise. Univariable and multivariable logistic regression and Cox proportional hazard models were used to calculate hazard ratios (HRs), respectively. The Youden index was used to calculate the cutoff values for nTMA and VAT in relation to 5-year, all-cause mortality. A *p* value < 0.05 was considered statistically significant, and all calculations were performed using R version 4.0.2. [26].

## 3. Results

The mean age within this cohort was 82.8 ± 5.95 years (IQR: 79.0–86.5), with 304 (52.1%) of the patients identified as female (Table 1). The association between age and an elevated risk of 5-year mortality was not statistically significant, as indicated by a HR of 1.023 (95% CI: 0.998–1.049; *p* = 0.069). Similarly, body mass index (BMI) did not exhibit a significant correlation with an increased 5-year mortality risk, revealing an HR of 1.005 (95% CI: 0.97–1.05; *p* = 0.8).

**Figure 2:**
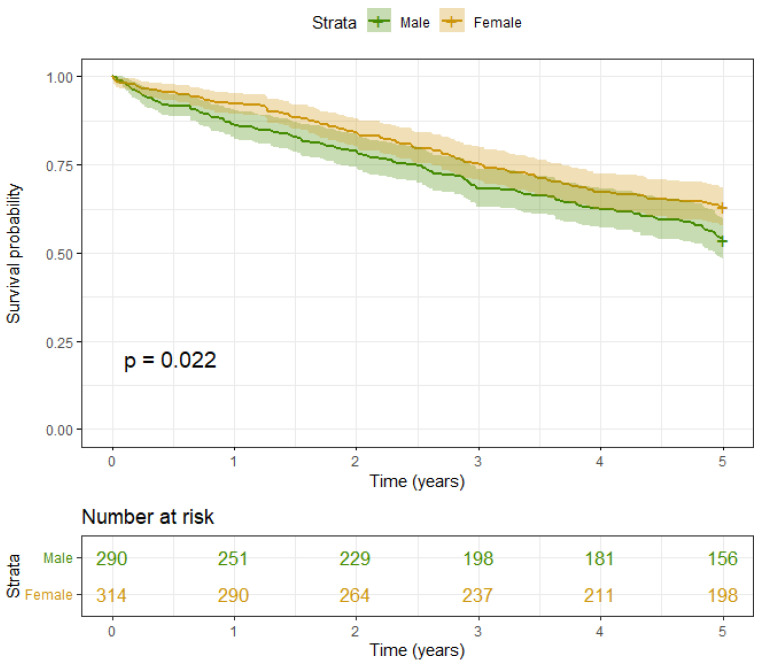

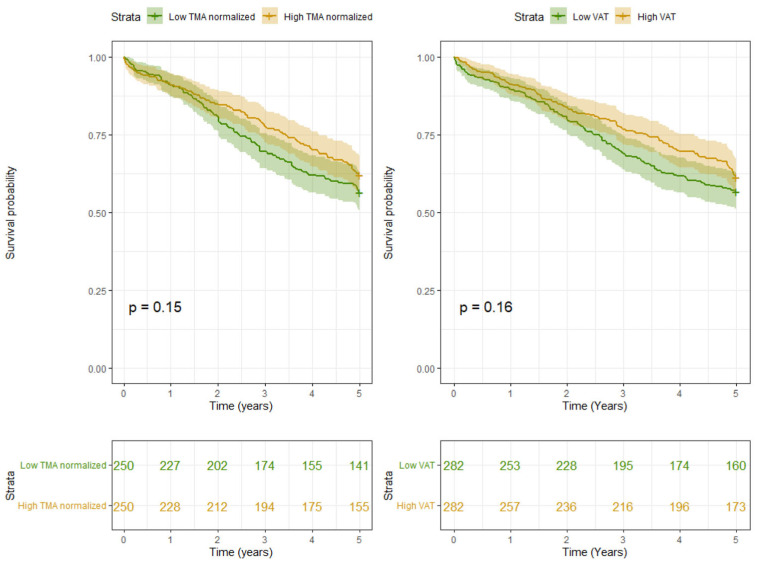
5-year survival curves, associated *p*-values from the log rank test and number at risk stratified according to gender.

Contrastingly, a notable difference in survival rates was identified between female and male patients, with female patients demonstrating a superior survival outcome (HR: 0.036, 95% CI: 0.003–0.39, *p* = 0.007) over the 5-year follow-up period (Table 2, Figure 2). Furthermore, a noteworthy interaction between female gender and nTMA was observed (HR: 1.048, 95%-CI: 1.013–1.084; *p* = 0.007) (Table 2).

In consideration of individual CT tissue parameters, the following results were ascertained:

(I) Muscle mass:

PMA exhibited no significant association with an increased 5-year mortality, as evidenced by a HR of 0.970 (95% CI: 0.921–1.026; *p* = 0.3). In contrast, nTMA demonstrated a statistically significant association with elevated 5-year, all-cause mortality, revealing an HR of 0.96 (95% CI: 0.927–0.997; *p* = 0.033).

**Figure 3:**
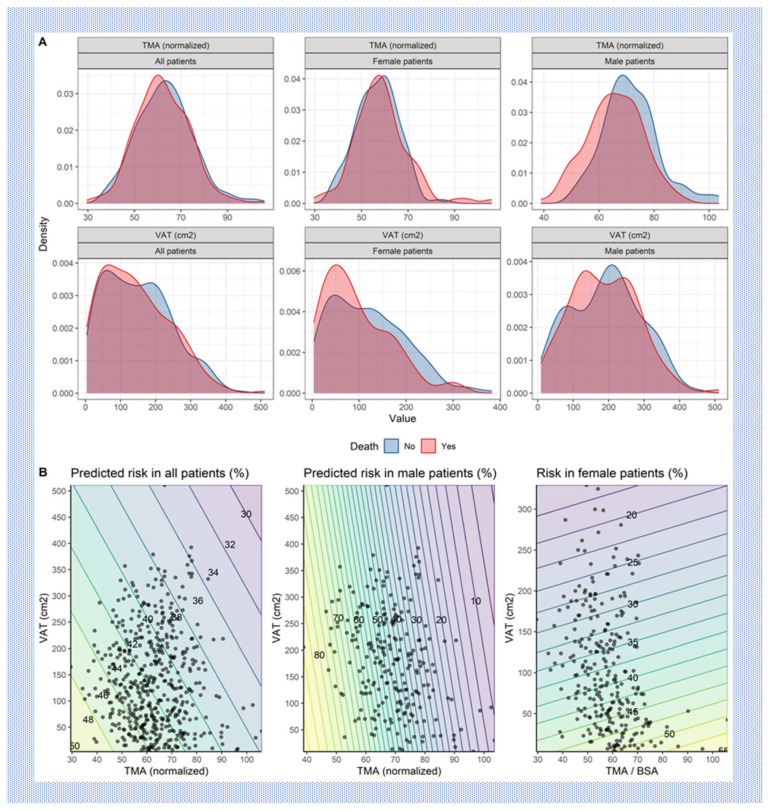
(**A**) Marginal distributions of total muscle area (TMA) and visceral adipose tissue (VAT) in surviving and deceased patients for the entire cohort and stratified according to gender. (**B**) Predicted 5-year mortality risk when considering TMA and VAT jointly.

When analyzing sex-specific outcomes, we observed a higher survival probability for the whole 5-year period, with *p* = 0.022 in female patients compared to male patients. (Table 2) (Figure 2). A protective effect of higher nTMA levels was observed in male patients (p_interaction_: sex × TMA (normalized) = 0.007) (Table 2). The marginal distributions of TMA and VAT in surviving and deceased patients for the entire cohort, as well as stratified according to gender, are presented in Figure 3.

(II) Fat mass:

Regarding VAT, no significant difference in survival was observed (HR 1.002, 95% CI: 0.99–1.015; *p* = 0.7). Similarly, when considering the influence of VAT on survival outcomes, gender did not exhibit a discernible effect, indicated by a HR of 0.99 (95% CI: 0.99–1.001; *p* = 0.2) (Table 2).

(III) Interaction of Muscle and Fat mass:

The combination of nTMA with VAT (cm^2^) was not associated with higher mortality and HR 1.00 (95% CI:1.00–1.00; *p* = 0.5) (Table 2). Also the gender-specific examination of the combined effect of nTMA and VAT did not exhibit a correlation with elevated mortality rates (Table 3).

## 4. Discussion

In the present study, we investigated various body composition parameters for the purpose of a comprehensive assessment of frailty using the obligatory CT scan before TAVR. Our study showed that low TMA, i.e., sarcopenia, was associated with higher 5-year all-cause mortality and was an additional tool for the comprehensive assessment of frailty. The effect of nTMA on 5-year all-cause mortality was gender-dependent. Here, the protective effect of higher nTMA levels was found in male patients (p_interaction_: sex × nTMA = 0.007). But PMA and VAT were not associated with higher 5-year mortality. Also, the combination of TMA and VAT was not a stronger parameter for lower survival.

In the existing body of literature, risk assessment predominantly revolves around the investigation of two primary tissues, namely muscle mass and fat mass. Additionally, comprehensive frailty assessments often incorporate further parameters, such as low bone mineral density. However, although low bone mineral density is significantly associated with an increased cardiovascular risk regarding the interaction of low bone mineral density and TAVR outcome, only spare data are available [27,28].

Regarding fat mass, subcutaneous fat and visceral adipose tissue have been investigated in several previous studies analyzing the effects on noncardiac and cardiac perioperative outcomes and survival [22,23]. For TAVR, a previous study demonstrated that VAT was not associated with significant differences, but patients with higher subcutaneous adipose tissue (SAT) had a significantly lower incidence of composite outcome and all-cause death compared with patients with lower SAT [23]. In another study, it was observed that a low volume of subcutaneous and visceral adipose tissue was associated with worse clinical outcomes than a high volume in patients undergoing TAVR [29]. Thus, with regard to VAT, there are conflicting data in the literature. Previous studies have also analyzed adipose tissue in non-cardiac procedures. Thus, in patients with locally advanced rectal cancer, a high visceral to subcutaneous adipose tissue ratio is the body composition parameter most strongly associated with poor early postoperative outcomes [30]. The ratio of visceral to subcutaneous fat also has a significant impact on postoperative complications in colorectal cancer [31].

In our cohort, VAT was not significantly associated with higher mortality. Even after a sex-specific analysis, no significant difference was found between a low and a high VAT value. Whether adipose tissue has different effects on outcome depending on cardiac or noncardiac interventions needs to be investigated in further studies.

Regarding muscle mass, valuable studies have evaluated PMA for the diagnosis of sarcopenia, as it is one of the key components of the frailty syndrome. In noncardiac surgery such as elective spine surgery, PMA was a predictor of the need for intensive care and postoperative blood transfusion [32]. Moreover, in patients with acute mesenteric ischemia, postoperative complications and 30-day mortality were lower in patients with low PMA [33]. Sarcopenia, defined by psoas muscle mass, was also an independent predictor of 2-year mortality, major complications, and severity of complications after major colorectal surgery [34]. PMA was an objective frailty assessment tool predicting early morbidity and mortality following spine surgery [21]. Also, in cardiac surgery low PMA was associated with a worse outcome [35,36]. Low PMA was also associated with an increased length of stay in older adults undergoing cardiac surgical procedures [37]. In cardiac procedures, PMA was described as a relevant predictor of a poor outcome. In patients undergoing TAVR, the PMA index significantly predicted 1-year risk-adjusted mortality and long-term mortality, as well as risk-adjusted severe morbidity, prolonged ventilation, length of stay, hospital discharge, and hospital costs [38]. But in the present study, low PMA was not associated with higher 5-year mortality. Nevertheless nTMA, which includes the total muscle mass of a given segment, was significantly associated with higher 5-year mortality. This shows the importance of nTMA measurement since it could be a stronger predictor for frailty assessment compared to PMA and should be performed regularly.

Further, in the present study the protective effect of higher nTMA levels was found in male patients. Gender differences in this regard need to be investigated in further studies.

## 5. Conclusions

In summary, the nTMA has emerged as a robust predictor of all-cause mortality, showcasing its potential as a novel and valuable supplementary tool in the realm of risk assessment. This parameter not only complements but enhances well-established frailty prediction tools, offering a more nuanced and comprehensive understanding of a patient’s health status.

The significance of nTMA extends beyond its role in frailty assessment, particularly in the context of patients undergoing TAVR. Its capacity to predict long-term survival post-TAVR highlights its clinical utility as a prognostic marker that transcends frailty considerations alone. This reinforces the notion that body composition parameters, specifically nTMA, encapsulate valuable insights into a patient’s overall health and resilience, influencing outcomes in a broader context.

As an adjunct to traditional frailty assessment tools, the inclusion of nTMA in risk stratification protocols holds promise in refining risk prediction models and optimizing patient care strategies. Its robust association with all-cause mortality underscores its potential as a pivotal metric in cardiovascular risk assessment, steering towards a more holistic and personalized approach in the evolving landscape of cardiovascular care.

## 6. Study Limitations

The primary limitation inherent in this study resides in the intricacies associated with the frailty syndrome. Although body composition parameters inherently tied to imaging provide valuable insights, they constitute only a partial aspect within the expansive realm of a comprehensive frailty assessment. Recognizing the multifaceted nature of frailty, it is imperative to emphasize that an exhaustive evaluation should extend beyond the singular consideration of imaging parameters. However, we assert that the integration of these objective measures, when judiciously combined with CT scans, can significantly enrich our understanding of frailty. This augmentation serves to complement traditional factors such as age, cognitive impairment, physical weakness, and occupational values, thereby contributing to a more holistic assessment of the intricate frailty landscape.

Furthermore, this study brings to light certain gender-specific disparities, introducing a layer of ambiguity into the findings. The rationale behind the observable impact of total muscle area on outcomes in males, in contrast to its less evident influence in females, lacks clarity. This gender-specific incongruity necessitates a deeper exploration in subsequent investigations to unravel the underlying mechanisms and elucidate potential nuances that may be influencing the observed outcomes. A thorough examination of gender-specific factors interacting with body composition parameters and frailty could refine risk stratification models and contribute to the development of more personalized healthcare strategies.

In essence, while acknowledging the valuable contributions of body composition parameters to our understanding of frailty, this study serves as a catalyst for ongoing research endeavors. The complexities surrounding frailty, especially in light of gender-specific variations, warrant continuous investigation to unravel the intricacies of this syndrome comprehensively. The pursuit of a more nuanced comprehension holds the potential to refine risk prediction models, optimize patient care strategies, and foster a more personalized approach to healthcare delivery in the context of frailty and related cardiovascular interventions.

## Figures and Tables

**Table 1:** Baseline Characteristics.

	All Patients *n* = 584
Gender:	
Male	280 (47.9%)
Female	304 (52.1%)
Age (years)	82.8 [79.0; 86.5]
Height (cm)	165 [158; 172]
Weight (kg)	71.0 [62.0; 83.0]
Body mass index (kg/m^2^)	25.6 [22.7; 29.7]
Diabetes mellitus [Yes]	156 (26.7%)
Arterial hypertension [Yes]	506 (86.6%)
Dyslipidemia [Yes]	391 (67.0%)
Chronic obstructive pulmonary disease [Yes]	61 (10.4%)
History of cerebrovascular accident [Yes]	71 (12.2%)
Transient ischemic attack [Yes]	31 (5.31%)
Coronary artery disease [Yes]	346 (59.2%)
History of myocardial infarction [Yes]	87 (14.9%)
Atrial fibrillation [Yes]	203 (34.8%)
Peripheral artery disease [Yes]	69 (11.8%)
History of cardiac surgery [Yes]	59 (10.1%)
Dyspnea [Yes]	578 (99.1%)
Body surface area (m^2^, Haycock)	1.81 [1.67; 1.99]
Visceral adipose tissue (cm^2^)	134 [68.5; 216]
Total muscle area (cm^2^)	110 [93.4; 131]
Total muscle area normalized by body surface area (-)	62.3 [54.5; 70.1]
Subcutaneous adipose tissue (cm^2^)	148 [106; 207]
Creatinine (µmol/L)	95.5 [77.0; 120]
Brain natriuretic peptide (pg/mL)	257 [108; 625]
Albumin (g/L)	34.0 [32.0; 36.0]
Mean gradient of the aortic valve (mmHg)	39.0 [28.0; 47.0]
Peak gradient of the aortic valve (mmHg)	63.0 [45.0; 78.0]
Aortic valve area (cm^2^)	0.70 [0.60; 0.90]
Indexed aortic valve area (cm^2^)	0.27 [0.21; 0.32]
Left ventricular ejection fraction (LVEF) (%)	60.0 [50.0; 65.0]
Logistic Euro Score	9.18 [5.83; 17.7]
Linear Euro Score	8.00 [6.00; 10.0]
Euro Score-II	3.67 [2.24; 6.66]
STS predicted risk of mortality	4.12 [2.90; 6.31]

**Table 2:** Regression coefficient and associated 95% confidence intervals and *p*-values for a multivariable logistic regression model and multivariable survival cox regression model.

	Cox Regression		
Characteristic	HR	95% CI	*p*-Value
Age (years)	1.023	0.998, 1.049	*p* = 0.069
Body mass index (kg/m^2^)	1.005	0.97, 1.046	*p* = 0.8
Sex:			
Female	0.036	0.003, 0.396	*p* = 0.007
TMA (normalized)	0.96	0.927, 0.997	*p* = 0.033
VAT (cm^2^)	1.002	0.99, 1.015	*p* = 0.7
Sex × TMA (normalized)			
Female × TMA (normalized)	1.048	1.013, 1.084	*p* = 0.007
Sex × VAT			
Female × VAT (cm^2^)	0.997	0.99, 1.001	*p* = 0.2
TMA (normalized) × VAT (cm^2^)	0.9999	0.9998, 1.	*P* = 0.5

**Table 3:** Gender-specific regression coefficient and associated 95% confidence intervals and *p*-values for multivariable survival cox regression model.

	Female Patients			Male Patients		
Characteristic	HR ^1^	95% CI ^1^	*p*-Value	HR ^1^	95% CI ^1^	*p*-Value
Age (years)	1.029	0.99, 1.070	*p* = 0.14	1.018	0.99, 1.052	*p* = 0.3
BMI (kg/m^2^)	1.003	0.948, 1.061	*p* = >0.9	1.006	0.95, 1.065	*p* = 0.8
TMA (normalized)	1.009	0.98, 1.038	*p* = 0.6	0.96	0.917, 0.999	*p* = 0.044
VAT (cm^2^)	1.001	0.98, 1.019	*p* = >0.9	1.001	0.99, 1.017	*p* = 0.9
TMA (normalized) × VAT (cm^2^)	0.9999	0.9996, 1.	*p* = 0.6	0.	0.9997, 1.	*p* = 0.7

^1^ HR = Hazard Ratio, CI = Confidence Interval, BMI = body mass index, TMA= total muscle area, VAT=visceral adipose tissue.

## Data Availability

The data underlying this article is available in the TAVR Register of Bern and will be shared on reasonable request to the corresponding author.

## Abbreviations

TAVR	Transcatheter aortic valve replacement
AS	Aortic valve stenosis
TMA	Total muscle area
nTMA	Normalized total muscle area
PMA	Psoas muscle area
VAT	Visceral adipose tissue

## References

[1] Mack M.J., Leon M.B., Thourani V.H., Makkar R., Kodali S.K., Russo M., Kapadia S.R., Malaisrie S.C., Cohen D.J., Pibarot P. (2019). Transcatheter Aortic-Valve Replacement with a Balloon-Expandable Valve in Low-Risk Patients. N. Engl. J. Med..

[2] Popma J.J., Deeb G.M., Yakubov S.J., Mumtaz M., Gada H., O’Hair D., Bajwa T., Heiser J.C., Merhi W., Kleiman N.S. (2019). Transcatheter Aortic-Valve Replacement with a Self-Expanding Valve in Low-Risk Patients. N. Engl. J. Med..

[3] Reardon M.J., Van Mieghem N.M., Popma J.J., Kleiman N.S., Søndergaard L., Mumtaz M., Adams D.H., Deeb G.M., Maini B., Gada H. (2017). Surgical or Transcatheter Aortic-Valve Replacement in Intermediate-Risk Patients. N. Engl. J. Med..

[4] Leon M.B., Smith C.R., Mack M.J., Makkar R.R., Svensson L.G., Kodali S.K., Thourani V.H., Tuzcu E.M., Miller D.C., Herrmann H.C. (2016). Transcatheter or Surgical Aortic-Valve Replacement in Intermediate-Risk Patients. N. Engl. J. Med..

[5] Dowling C., Kondapally Seshasai S.R., Firoozi S., Brecker S.J. (2020). Transcatheter aortic valve replacement versus surgery for symptomatic severe aortic stenosis: A reconstructed individual patient data meta-analysis. Catheter. Cardiovasc. Interv..

[6] Makkar R.R., Thourani V.H., Mack M.J., Kodali S.K., Kapadia S., Webb J.G., Yoon S.-H., Trento A., Svensson L.G., Herrmann H.C. (2020). Five-Year Outcomes of Transcatheter or Surgical Aortic-Valve Replacement. N. Engl. J. Med..

[7] Schenker C., Wertli M.M., Räber L., Haynes A.G., Chiolero A., Rodondi N., Panczak R., Aujesky D. (2024). Regional variation and temporal trends in transcatheter and surgical aortic valve replacement in Switzerland: A population-based small area analysis. PLoS ONE.

[8] Grossman Y., Barbash I.M., Fefer P., Goldenberg I., Berkovitch A., Regev E., Fink N., Ben-Zekry S., Brodov Y., Kogan A. (2017). Addition of albumin to Traditional Risk Score Improved Prediction of Mortality in Individuals Undergoing Transcatheter Aortic Valve Replacement. J. Am. Geriatr. Soc..

[9] Afilalo J., Lauck S., Kim D.H., Lefèvre T., Piazza N., Lachapelle K., Martucci G., Lamy A., Labinaz M., Peterson M.D. (2017). Frailty in Older Adults Undergoing Aortic Valve Replacement: The FRAILTY-AVR Study. J. Am. Coll. Cardiol..

[10] Søndergaard L., Kirk B.H., Jørgensen T.H. (2018). Frailty: An Important Measure in Patients Considered for Transcatheter Aortic Valve Replacement. JACC Cardiovasc. Interv..

[11] Ilic I., Faron A., Heimann M., Potthoff A.-L., Schäfer N., Bode C., Borger V., Eichhorn L., Giordano F.A., Güresir E. (2021). Combined Assessment of Preoperative Frailty and Sarcopenia Allows the Prediction of Overall Survival in Patients with Lung Cancer (NSCLC) and Surgically Treated Brain Metastasis. Cancers.

[12] Bentov I., Kaplan S.J., Pham T.N., Reed M.J. (2019). Frailty assessment: From clinical to radiological tools. Br. J. Anaesth..

[13] Flexman A.M., Street J., Charest-Morin R. (2019). The impact of frailty and sarcopenia on patient outcomes after complex spine surgery. Curr. Opin. Anaesthesiol..

[14] Kołodziejska K., Witowski J., Tylec P., Grochowska A., Przytuła N., Lis M., Pędziwiatr M., Rubinkiewicz M. (2022). Radiological Features for Frailty Assessment in Patients Requiring Emergency Laparotomy. J. Clin. Med..

[15] de Bree R., Meerkerk C.D.A., Halmos G.B., Mäkitie A.A., Homma A., Rodrigo J.P., López F., Takes R.P., Vermorken J.B., Ferlito A. (2022). Measurement of Sarcopenia in Head and Neck Cancer Patients and Its Association with Frailty. Front. Oncol..

[16] Okamura H., Kimura N., Mieno M., Yuri K., Yamaguchi A. (2020). Preoperative sarcopenia is associated with late mortality after off-pump coronary artery bypass grafting. Eur. J. Cardiothorac. Surg..

[17] Canales C., Mazor E., Coy H., Grogan T.R., Duval V., Raman S., Cannesson M., Singh S.P. (2022). Preoperative Point-of-Care Ultrasound to Identify Frailty and Predict Postoperative Outcomes: A Diagnostic Accuracy Study. Anesthesiology.

[18] Meng N.H., Li C.I., Liu C.S., Lin W.-Y., Lin C.-H., Chang C.-K., Li T.-C., Lin C.-C. (2015). Sarcopenia Defined by Combining Height- and Weight-Adjusted Skeletal Muscle Indices is Closely Associated with Poor Physical Performance. J. Aging Phys. Act..

[19] McIsaac D.I. (2022). Preoperative Frailty Assessment: An Opportunity to Add Value to Perioperative Care. Anesthesiology.

[20] Mamane S., Mullie L., Piazza N., Martucci G., Morais J., Vigano A., Levental M., Nelson K., Lange R., Afilalo J. (2016). Psoas Muscle Area and All-Cause Mortality After Transcatheter Aortic Valve Replacement: The Montreal-Munich Study. Can. J. Cardiol..

[21] Saji M., Lim D.S., Ragosta M., LaPar D.J., Downs E., Ghanta R.K., Kern J.A., Dent J.M., Ailawadi G. (2016). Usefulness of Psoas Muscle Area to Predict Mortality in Patients Undergoing Transcatheter Aortic Valve Replacement. Am. J. Cardiol..

[22] Mok M., Allende R., Leipsic J., Altisent O.A.-J., del Trigo M., Campelo-Parada F., DeLarochellière R., Dumont E., Doyle D., Côté M. (2016). Prognostic Value of Fat Mass and Skeletal Muscle Mass Determined by Computed Tomography in Patients Who Underwent Transcatheter Aortic Valve Implantation. Am. J. Cardiol..

[23] Okuno T., Koseki K., Nakanishi T., Ninomiya K., Tomii D., Tanaka T., Sato Y., Osanai A., Sato K., Koike H. (2018). Prognostic Impact of Computed Tomography-Derived Abdominal Fat Area on Transcatheter Aortic Valve Implantation. Circ. J..

[24] Stortecky S., Franzone A., Heg D., Tueller D., Noble S., Pilgrim T., Jeger R., Toggweiler S., Ferrari E., Nietlispach F. (2019). Temporal trends in adoption and outcomes of transcatheter aortic valve implantation: A SwissTAVI Registry analysis. Eur. Heart J. Qual. Care Clin. Outcomes.

[25] Tomii D., Okuno T., Heg D., Lanz J., Praz F., Stortecky S., Windecker S., Pilgrim T. (2022). Validation of the VARC-3 Technical Success Definition in Patients Undergoing TAVR. JACC Cardiovasc. Interv..

[26] R Core Team (2020). R: A Language and Environment for Statistical Computing.

[27] Rodríguez A.J., Scott D., Hodge A., English D.R., Giles G.G., Ebeling P.R. (2017). Associations between hip bone mineral density, aortic calcification and cardiac workload in community-dwelling older Australians. Osteoporos. Int..

[28] Shi L., Yu X., Pang Q., Chen X., Wang C. (2022). The associations between bone mineral density and long-term risks of cardiovascular disease, cancer, and all-cause mortality. Front. Endocrinol..

[29] Shibata K., Yamamoto M., Yamada S., Kobayashi T., Morita S., Kagase A., Tokuda T., Shimura T., Tsunaki T., Tada N. (2021). Clinical Outcomes of Subcutaneous and Visceral Adipose Tissue Characteristics Assessed in Patients Underwent Transcatheter Aortic Valve Replacement. CJC Open.

[30] Bocca G., Mastoridis S., Yeung T., James D.R.C., Cunningham C. (2022). Visceral-to-subcutaneous fat ratio exhibits strongest association with early post-operative outcomes in patients undergoing surgery for advanced rectal cancer. Int. J. Color. Dis..

[31] He A.Q., Li C.Q., Zhang Q., Liu T., Liu J., Liu G. (2021). Visceral-to-Subcutaneous Fat Ratio Is a Potential Predictor of Postoperative Complications in Colorectal Cancer. Med. Sci. Monit..

[32] Pernik M.N., Hicks W.H., Akbik O.S., Nguyen M.L., Luu I., Traylor J.I., Deme P.R., Dosselman L.J., Hall K., Wingfield S.A. (2022). Psoas Muscle Index as a Predictor of Perioperative Outcomes in Geriatric Patients Undergoing Spine Surgery. Global Spine J..

[33] Miao S.L., Ye X.N., Lin T.T., Qiu Y.-H., Huang J.-Y., Zheng X.-W., Chen F.-F. (2022). The psoas muscle density as a predictor of postoperative complications and 30-day mortality for acute mesenteric ischemia patients. Abdom. Radiol..

[34] Batista A.F.R., Petty D., Fairhurst C., Davies S. (2023). Psoas muscle mass index as a predictor of long-term mortality and severity of complications after major intra-abdominal colorectal surgery—A retrospective analysis. J. Clin. Anesth..

[35] Balsam L.B. (2018). Psoas muscle area: A new standard for frailty assessment in cardiac surgery?. J. Thorac. Dis..

[36] Paknikar R., Friedman J., Cron D., Deeb G.M., Chetcuti S., Grossman P.M., Wang S., Englesbe M., Patel H.J. (2016). Psoas muscle size as a frailty measure for open and transcatheter aortic valve replacement. J. Thorac. Cardiovasc. Surg..

[37] Zuckerman J., Ades M., Mullie L., Trnkus A., Morin J.-F., Langlois Y., Ma F., Levental M., Morais J.A., Afilalo J. (2017). Psoas Muscle Area and Length of Stay in Older Adults Undergoing Cardiac Operations. Ann. Thorac. Surg..

[38] Hawkins R.B., Mehaffey J.H., Charles E.J., Kern J.A., Lim D.S., Teman N.R., Ailawadi G. (2018). Psoas Muscle Size Predicts Risk-Adjusted Outcomes After Surgical Aortic Valve Replacement. Ann. Thorac. Surg..

